# Genotype‐Dependent Modulation of Macrophage Apoptosis and T Lymphocyte Responses by Duck Tembusu Virus Potentially Contributes to Genotype‐Specific Immunopathogenesis

**DOI:** 10.1155/tbed/3076122

**Published:** 2025-12-23

**Authors:** Teerawut Nedumpun, Kanana Rungprasert, Benchaphorn Limcharoen, Aunyaratana Thontiravong

**Affiliations:** ^1^ Department of Veterinary Microbiology, Faculty of Veterinary Science, Chulalongkorn University, Pathumwan, Bangkok, 10330, Thailand, chula.ac.th; ^2^ Center of Excellence for Emerging and Re-emerging Infectious Diseases in Animals (CUEIDAs), Faculty of Veterinary Science, Chulalongkorn University, Pathumwan, Bangkok, 10330, Thailand, chula.ac.th; ^3^ Center of Excellence for Veterinary Clinical Stem Cells and Bioengineering, Chulalongkorn University, Pathumwan, Bangkok, 10330, Thailand, chula.ac.th; ^4^ Department of Anatomy, Faculty of Veterinary Science, Chulalongkorn University, Pathumwan, Bangkok, 10330, Thailand, chula.ac.th; ^5^ Center of Excellence in Animal Vector-Borne Diseases, Veterinary Parasitology Unit, Department of Pathology, Faculty of Veterinary Science, Chulalongkorn University, Pathumwan, Bangkok, 10330, Thailand, chula.ac.th; ^6^ Center of Excellence of Systems Microbiology, Faculty of Medicine, Chulalongkorn University, Pathumwan, Bangkok, 10330, Thailand, chula.ac.th

**Keywords:** apoptosis, duck Tembusu virus, macrophage, T lymphocyte

## Abstract

Duck Tembusu virus (DTMUV) is an emerging avian pathogenic flavivirus that causes neurological disorders and acute egg drop syndrome in ducks. Multiple genotypes of DTMUV, including clusters 1, 2, and 3, have been identified, and recent evidence suggests a correlation between viral genotype and disease severity. Our recent study demonstrated that DTMUV cluster 2.1 induces more severe clinical disease in ducks than cluster 1; however, the underlying mechanisms responsible for this difference remain unclear. Macrophages are key target cells for DTMUV replication and play a crucial role during the early stage of viral pathogenesis. Therefore, we hypothesized that distinct DTMUV genotypes may differentially regulate macrophage apoptosis, thereby influencing viral replication and modulating T lymphocyte responses. To investigate this, we developed an in vitro protocol to generate duck monocyte‐derived macrophages (MDMs) and assessed their response to infection with two different DTMUV genotypes: cluster 1 and cluster 2.1. Our results showed that DTMUV cluster 1 infection significantly increased macrophage apoptosis and reduced cell viability. In contrast, DTMUV cluster 2.1‐infected MDM exhibited higher cell viability and fewer apoptotic cells. Correspondingly, viral titers in supernatants from DTMUV cluster 2.1‐infected cultures were significantly higher than those from cluster 1. Furthermore, culture with DTMUV cluster 2.1 resulted in enhanced proliferation of Th (CD3^+^CD4^+^BrdU^+^) and cytotoxic T lymphocyte (CTL; CD3^+^CD8^+^BrdU^+^) lymphocytes in both naïve peripheral blood mononuclear cells (PBMCs) and macrophage‐peripheral blood lymphocytes (PBLs) co‐culture systems. These results suggest that the inhibition of apoptosis in DTMUV cluster 2.1‐infected macrophages may enhance antigen‐specific T lymphocyte responses. Overall, our findings reveal a genotype‐dependent mechanism by which DTMUV modulates macrophage survival, thereby influencing both viral replication and T cell activation, and ultimately contributing to genotype‐specific immunopathogenesis. These insights deepen our understanding of DTMUV immunopathogenesis and may inform the development of more effective genotype‐targeted vaccination and disease control strategies.

## 1. Introduction

Duck Tembusu virus (DTMUV) is an emerging avian pathogenic flavivirus that causes severe neurological disorders and acute egg drop syndrome in ducks and other avian species, including geese and chickens [1, 2]. Several studies have consistently shown that DTMUV primarily induces growth retardation and severe neurological signs in young ducks, and a marked reduction in egg production in laying ducks [3, 4]. Currently, DTMUV is widely distributed and has become endemic in duck populations across Asia, leading to substantial economic losses in the duck‐producing industry.

DTMUV is an enveloped, single‐stranded, positive‐sense RNA virus classified as a new genotype of Tembusu virus (TMUV) within the genus *Flavivirus*, family Flaviviridae [1, 5]. Currently, DTMUV is genetically divided into three distinct clusters: cluster 1, cluster 2 (subclusters 2.1 and 2.2), and cluster 3 [6]. All three clusters have been reported across Asia, showing distinct geographical distributions [6]. Interestingly, recent studies have demonstrated that, in addition to genetic divergence, variations in pathogenicity and disease severity also exist among DTMUV clusters [7–9]. We recently demonstrated that infection with DTMUV cluster 2.1 resulted in more rapid viremia, higher viral loads in target organs (brain and spleen), and more severe neurological signs compared with infection with DTMUV cluster 1 [7]. However, the immunological mechanisms underlying these differences remain largely unknown.

It is well established that DTMUV primarily targets macrophages during the early phase of infection to facilitate viral replication [10]. As key players in the innate immune system, macrophages not only initiate antiviral responses but also shape the magnitude and quality of adaptive immunity, particularly T lymphocyte activation and differentiation [11–13]. Several flaviviruses possess the ability to modulate macrophage function, a strategy often associated with immune evasion and disease progression in the host [13–16]. Among these strategies, induction of host cell apoptosis is a well‐recognized mechanism by which flaviviruses suppress early antiviral responses and facilitate viral persistence [17, 18]. Notably, previous studies have demonstrated that DTMUV induces apoptosis in duck fibroblast and embryonic cells [19, 20], raising questions about how this process affects duck macrophage survival and, in turn, modulates downstream T lymphocyte responses. Given that distinct DTMUV genotypes are associated with variations in clinical severity, we hypothesized that different genotypes may differentially regulate macrophage apoptosis, ultimately affecting T lymphocyte responses, and contributing to genotype‐specific immunopathogenesis. In this study, we investigated the effects of two DTMUV genotypes, cluster 1 and cluster 2.1, on macrophage apoptosis and T lymphocyte proliferation. This study aims to enhance our understanding of host‐pathogen interactions during DTMUV infection and provide foundational immunological insights that may inform the development of more effective, genotype‐targeted control strategies, including vaccine refinement.

## 2. Materials and Methods

### 2.1. Virus and Mock

Two DTMUV genotypes were used in this study: cluster 1 strain DK/TH/CU‐DTMUV2007 (GenBank accession no. MF621927) and cluster 2.1 strain DK/TH/CU‐1 (GenBank accession no. KR061333). Both viruses were propagated in baby hamster kidney (BHK‐21) cells. Following propagation, the cell culture supernatants were harvested, clarified by centrifugation, and subsequently filtered through 0.45 μm membrane filters. The viral titers were determined by conventional infectious virus titration in BHK‐21 cells, as previously described [21]. A mock control was prepared using lysates from uninfected BHK‐21 cells processed under identical condition as virus preparations. Both virus stocks and mock controls were aliquoted and stored in −80°C until use.

### 2.2. Isolation of Duck Peripheral Blood Mononuclear Cells (PBMCs), Peripheral Blood Lymphocytes (PBLs), and Generation of Duck Monocyte‐Derived Macrophages (MDMs)

PBMC were isolated from heparinized whole blood of healthy ducks using density gradient centrifugation with Isoprep separation medium (Robbins Scientific Co., CA, USA), according to the manufacturer’s instructions. Isolated cells were resuspended in Iscove’s Modified Dulbecco’s Medium (IMDM) supplemented with 5% fetal bovine serum (FBS). A total of 5 × 10^6^ cells were seeded into culture plates and incubated at 37°C in a humidified atmosphere containing 5% CO_2_ for 2 h. Following incubation, nonadherent cells, referred to as PBLs, were gently collected and cryopreserved in liquid nitrogen for later use. The remaining adherent monocyte population (~5% of total PBMC; ~2.5 × 10^5^ cells) was cultured in Dulbecco’s modified eagle medium (DMEM) supplemented with 20% autologous duck serum to induce macrophage differentiation. Flow cytometric analysis showed that the monocytes were negative for surface markers duck CD3 and duck IgY (data not shown), confirming the absence of T and B lymphocyte contamination. Cultures were maintained at 37°C with 5% CO_2_ for 5 days, with 50% of the medium replaced every 2 days using fresh DMEM with duck serum. The macrophage phenotype was confirmed by morphological evaluation under light microscopy. The resulting MDM were subsequently used for in vitro activation assays. All animal procedures were conducted under the approval of Faculty of Veterinary Science, Chulalongkorn University Animal Care and Use Committee (Approval Number 2531061).

### 2.3. In Vitro Activation for Assessing Macrophage Apoptosis

The duck MDM (*n* = 5) were infected with DTMUV cluster 1, cluster 2.1, or mock control at a multiplicity of infection (MOI) of 0.1 and incubated at 37°C with 5% CO_2_ for 48 h. DTMUV infection was confirmed by immunocytochemistry (ICC) using flavivirus‐specific antibodies (EMD Millipore Corporation, CA, USA), as described previously [22]. To examine the stage of virus‐induced apoptosis, MDM were harvested after 24 and 48 h of culture. The cells were washed with phosphate‐buffered saline (PBS) and stained with annexin V and propidium iodide (PI) using a commercial apoptosis detection kit (BioLegend, USA), following the manufacturer’s instructions and previously described protocols [23]. Flow cytometric analysis was performed to quantify apoptotic cells and differentiate among living cells (Annexin V^*−*^/PI^−^), early apoptosis (Annexin V^+^/PI^−^), apoptosis (Annexin V^+^/PI^+^), and dead cells (Annexin V^*−*^/PI^+^). Data were analyzed to compare the proportion of apoptotic MDM between the different DTMUV genotype‐infected and mock‐treated groups.

### 2.4. Assessment of DTMUV‐Specific T Lymphocyte Proliferation

Duck PBMC (*n* = 5) were incubated in complete RPMI‐1640 medium supplemented with 10 μM BrdU (BioLegend, USA) and infected with DTMUV cluster 1, cluster 2.1, or mock control at an MOI of 0.1. Cultures were maintained at 37°C in a 5% CO_2_ incubator for 72 h. In a parallel experiment, duck MDM (*n* = 5) were infected with the same DTMUV genotypes or mock control at an MOI of 0.1 and incubated for 24 h. Following incubation, the cultured MDM were co‐cultured with autologous duck PBL in BrdU‐containing RPMI medium for 72 h. Cells from both experimental conditions were harvested and stained to evaluate T lymphocyte proliferation. Surface markers were detected using anti‐human CD3ε‐Alexa Fluor 647 (clone CD3‐12, rat IgG1), anti‐duck CD4 (clone Du CD4‐2, mouse IgG2a), and anti‐duck CD8α (clone Du CD8‐1, mouse IgG2b), all from Bio‐Rad (CA, USA). After washing, isotype‐specific secondary antibodies, including goat anti‐mouse IgG2a‐FITC and goat anti‐mouse IgG2b‐PE/Cy7 (Southern Biotech, AL, USA), were added and incubated for 30 min at 4°C in the dark. For BrdU detection, cells were fixed, permeabilized, and stained with anti‐BrdU‐PE (1:100 dilution; BioLegend, USA) in permeabilization solution (BD Biosciences, CA, USA) for 30 min at 4°C. Fluorescence minus one (FMO) and isotype control antibodies were included to define gating strategies and background levels. Samples were acquired on a CytoFlex LX flow cytometer (Beckman Coulter, CA, USA), collecting a minimum of 5 × 10^4^ events per sample, and data were analyzed using FlowJo software (Tree Star Inc., OR, USA).

### 2.5. Viral RNA Extraction and Quantitative Real‐Time RT‐PCR (qRT‐PCR)

DTMUV RNA loads in supernatants from the indicated culture systems were quantified using a DTMUV envelope (E) gene‐specific qRT‐PCR assay, as previously described [3]. Briefly, viral RNA was extracted from culture supernatants (MDM culture) using the QIAamp Viral RNA Mini Kit (Qiagen, Hilden, Germany), following the manufacturer’s instructions. A total of 50 ng of extracted RNA was reverse‐transcribed into cDNA using random hexamer primers and the ImProm‐II Reverse Transcription System (Promega, WI, USA) in accordance with the manufacturer’s protocol. Quantitative real‐time PCR was subsequently performed using the TaqMan Fast Advanced Master Mix (Applied Biosystems, TX, USA). Viral RNA copy numbers were determined by absolute quantification based on a standard curve generated from a recombinant plasmid containing the DTMUV E gene. Final viral loads were normalized to 50 ng of total RNA. All standards and test samples were run in triplicate.

### 2.6. Statistical Analysis

Data were tested for normality using the Shapiro–Wilk test. Comparisons between two groups of study were analyzed using a two‐tailed unpaired Student’s *t*‐test. Correlation analyses between DTMUV load and the frequency of each apoptotic phenotype were performed using Pearson’s correlation test. All statistical analyses were performed using GraphPad Prism (GraphPad Software Inc., La Jolla, CA, USA). A *p*‐value of < 0.05 was considered statistically significant.

## 3. Results

### 3.1. Generation and Morphological Characterization of Duck MDM

To establish in vitro culture of immature duck MDM, PBMC were seeded in IMDM medium, and after 2 h of incubation, the adherent cells were designated as duck monocytes (Figure 1A). At day 0, duck monocytes displayed a round to oval morphology under light microscopy. Following 5 days of culture in MDM medium supplemented with 5% autologous duck serum, the cells differentiated into macrophages, characterized by increased cell size and an elongated morphology (Figure 1B). These duck MDM were subsequently used for downstream experiment.

Figure 1Generation and morphological characterization of duck monocyte‐derived macrophages (MDM). (A) The duck PBMC were isolated and incubated with IMDM supplemented 5% FBS for 2 h. Adherent cells, identified as monocytes, were subsequently cultured in DMEM containing autologous duck serum. (B) Morphological characteristics of duck MDM were observed by light microscopy on Day 5 of cultivation compared with Day 0.

**Figure:**
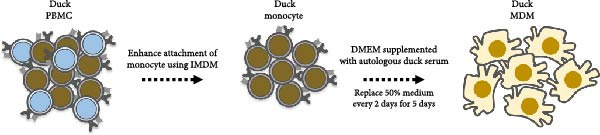
(A)

**Figure:**
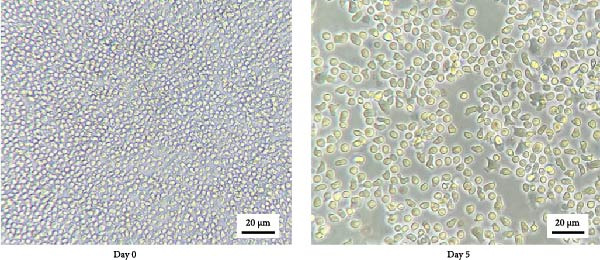
(B)

### 3.2. Low Apoptosis Induced by DTMUV Cluster 2.1 Infection Correlates With Increased DTMUV RNA Levels

To investigate the effect of DTMUV infection on apoptosis, duck MDM were in vitro cultured with DTMUV cluster 1 or cluster 2.1, and apoptotic cell populations were examined. The gating strategy for apoptotic stages is shown in Figure 2A. Living cells, early apoptotic cells, apoptotic cells, and dead cells, were classified as annexin V^*−*^/PI^-^, annexin V^+^/PI^−^, annexin V^+^/PI^+^, and annexin V^−^/PI^+^, respectively. At 48 h postinoculation, DTMUV cluster 1 infection decreased the proportion of living cells and increased early apoptotic cells compared with the mock control. In contrast, DTMUV cluster 2.1 infection maintained a higher proportion of living cells and induced fewer apoptotic cells than cluster 1 infection (Figure 2B, C). These findings indicate that DTMUV cluster 2.1 induces a weaker apoptotic response in duck MDM compared to cluster 1.

Figure 2Inhibition of apoptosis in duck MDM by DTMUV cluster 2.1. (A) Gating strategy for characterizing MDM apoptotic phenotypes: living cells (annexin V^*−*^PI^−^), early apoptotic cells (annexin V^+^PI^−^), apoptotic cells (annexin V^+^PI^+^), and dead cells (annexin V^*−*^PI^+^). (B, C) Percentages of each subpopulation were compared among cultures with DTMUV cluster 1, cluster 2.1, or mock (control) at (B) 24 and (C) 48 h postinoculation. Duck MDM were inoculated in vitro with DTMUV cluster 1, cluster 2.1 (0.1 MOI), or mock (*n* = 5 per treatment) for assessing macrophage apoptosis at indicated time. The apoptotic phenotypes were subsequently characterized by immunofluorescent staining.  ^∗^ indicates significant difference between treatments.

**Figure:**
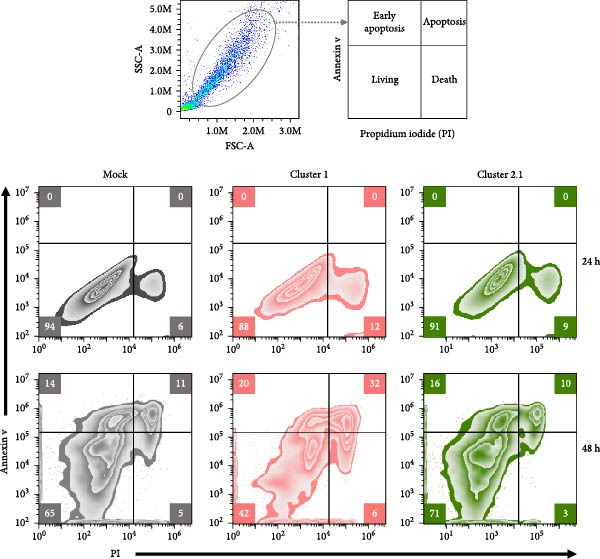
(A)

**Figure:**
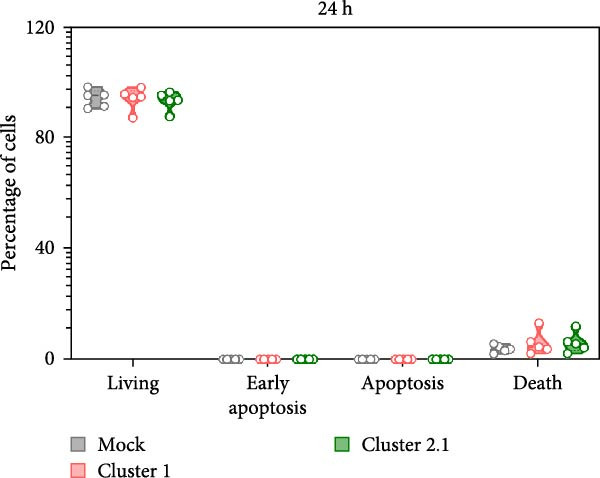
(B)

**Figure:**
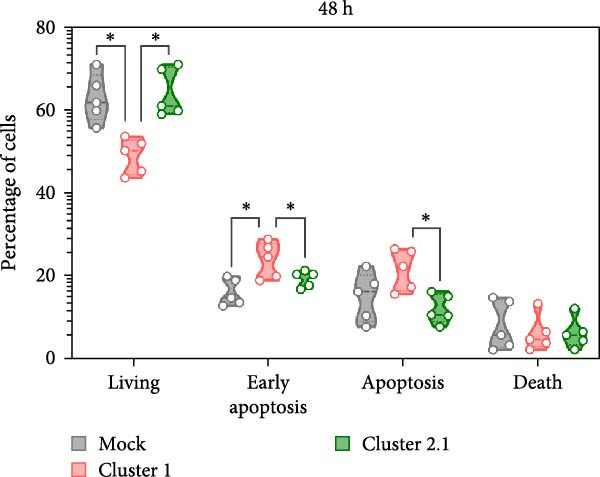
(C)

DTMUV infection in duck MDM was confirmed by ICC, with both strains clearly detected at 48 h postinoculation (Figure 3A). Quantification of DTMUV RNA load in culture supernatants by qRT‐PCR revealed increased viral loads following infection with both DTMUV strains. Notably, cultures infected with cluster 2.1 exhibited significantly higher viral RNA levels compared to cluster 1 (Figure 3B). Analysis of apoptotic responses further revealed that higher viral loads were positively correlated with increased numbers of viable duck MDM in cultures infected with both strains (Figure 3C,D). Interestingly, in cluster 2.1‐infected cultures, a reduction of early apoptotic cells was particularly associated with higher DTMUV loads (Figure 3D). Collectively, these findings indicate that DTMUV cluster 2.1 effectively inhibits apoptosis and promotes cell survival, thereby facilitating enhanced viral replication in duck MDM.

Figure 3Increased DTMUV RNA load in the supernatant of duck MDM infected with cluster 2.1 is associated with reduced early apoptosis. (A) DTMUV infection was confirmed by immunocytochemistry (ICC), with strong red staining indicating DTMUV‐positive cells. (B) Significantly higher levels of DTMUV RNA were detected in supernatants from cluster 2.1‐infected MDM compared with cluster 1. (C, D) Correlation analyses revealed that higher DTMUV loads were associated with increased frequencies of living cells and reduced frequencies of early apoptotic cells. Duck MDM were cultured with DTMUV cluster 1, cluster 2.1 (0.1 MOI), or mock (*n* = 5 per treatment) for 48 h. Supernatants were collected for qRT‐PCR analysis of DTMUV RNA load.  ^∗^ indicates significant difference between treatments or correlation analyses.

**Figure:**
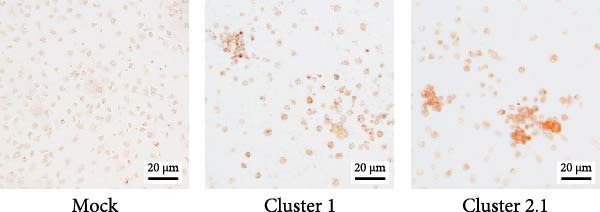
(A)

**Figure:**
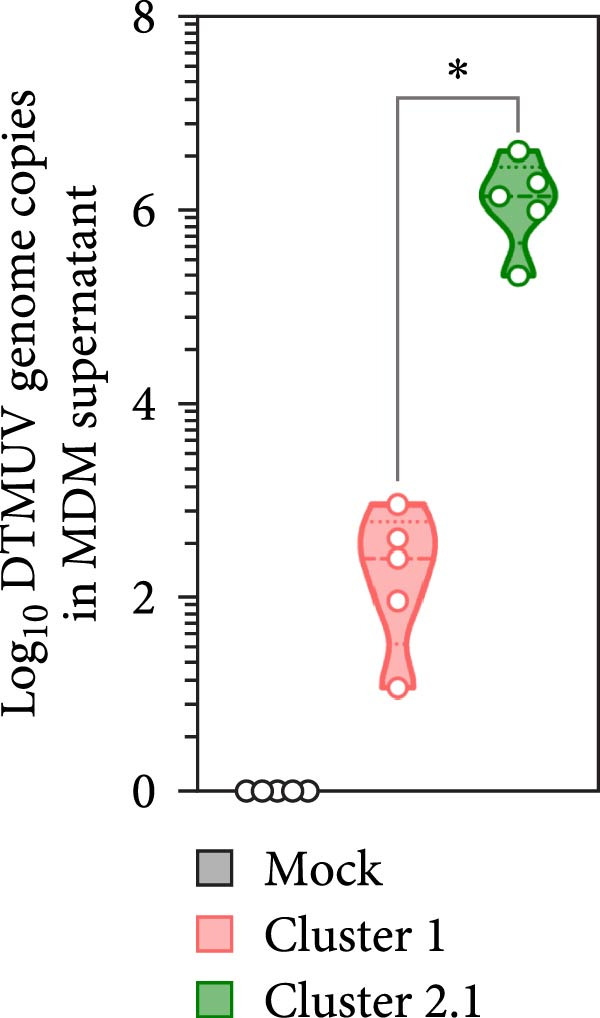
(B)

**Figure:**
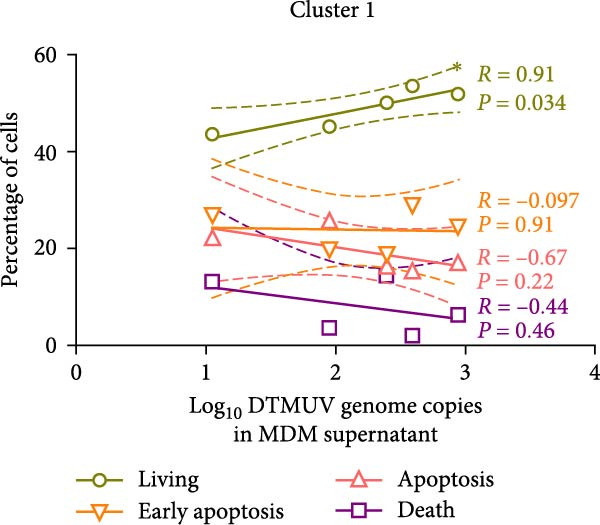
(C)

**Figure:**
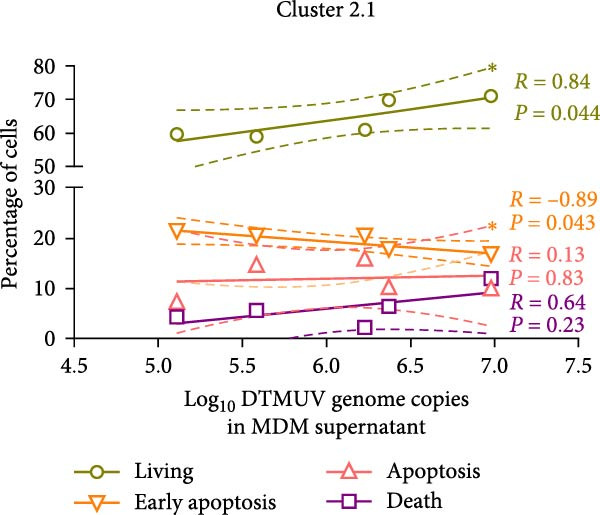
(D)

### 3.3. Enhanced DTMUV‐Specific T Lymphocyte Proliferation in Duck PBMC Cultured With DTMUV Cluster 2.1

Macrophages are known as efficient antigen‐presenting cells (APCs) that play a crucial role in activating antigen‐specific T lymphocyte responses. To preliminarily evaluate the impact of different DTMUV genotypes on duck cellular immunity, naïve duck PBMC were co‐cultured with DTMUV cluster 1 or cluster 2.1. Proliferating T lymphocyte subsets were identified using in vitro BrdU incorporation assay, with T helper (Th) lymphocytes defined as CD3^+^CD4^+^BrdU^+^ and cytotoxic T lymphocytes (CTLs) defined as CD3^+^CD8^+^BrdU^+^ (Figure 4A). The overall frequencies of Th and CTL were not significantly different among cultures (Figure 4B). However, compared with mock controls, cluster 2.1 infection markedly enhanced proliferation of both Th (Figure 4C) and CTL (Figure 4D) subpopulations, whereas cluster 1 did not induce such responses. These initial findings from PBMC cultures indicate that cluster 2.1 elicits a stronger DTMUV‐specific cellular immune response. This suggests that different DTMUV genotypes may differentially modulate APCs, thereby shaping the strength of T lymphocyte responses.

Figure 4Enhanced DTMUV‐specific T lymphocyte proliferation in duck PBMC cultured with DTMUV cluster 2.1. (A) Gating strategy used to identify proliferating T lymphocyte subpopulations in cultures of duck PBMC: T helper (Th) lymphocytes (CD3^+^CD4^+^BrdU^+^) and cytotoxic T lymphocytes (CTL) (CD3^+^CD8^+^BrdU^+^). (B) Overall frequencies of T lymphocyte subpopulations showed no significant differences across treatment groups. (C, D) Compared with cluster 1, cultures with DTMUV cluster 2.1 induced significantly higher proliferation of both Th and CTL subpopulations. Duck PBMC were cultured with DTMUV cluster 1, cluster 2.1 (0.1 MOI), or mock (*n* = 5 per treatment) for 72 h, followed by immunofluorescent staining to assess proliferative phenotypes.  ^∗^ indicates significant difference between treatments.

**Figure:**
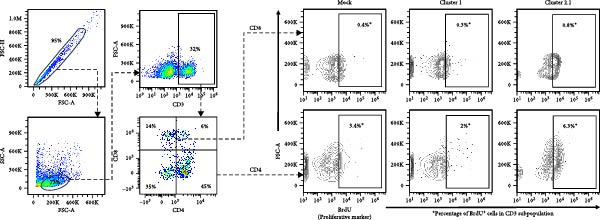
(A)

**Figure:**
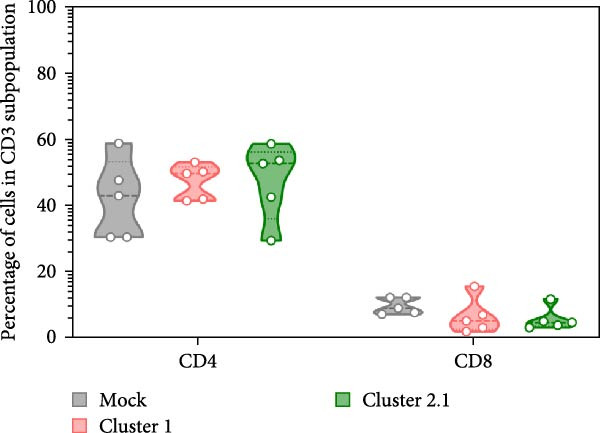
(B)

**Figure:**
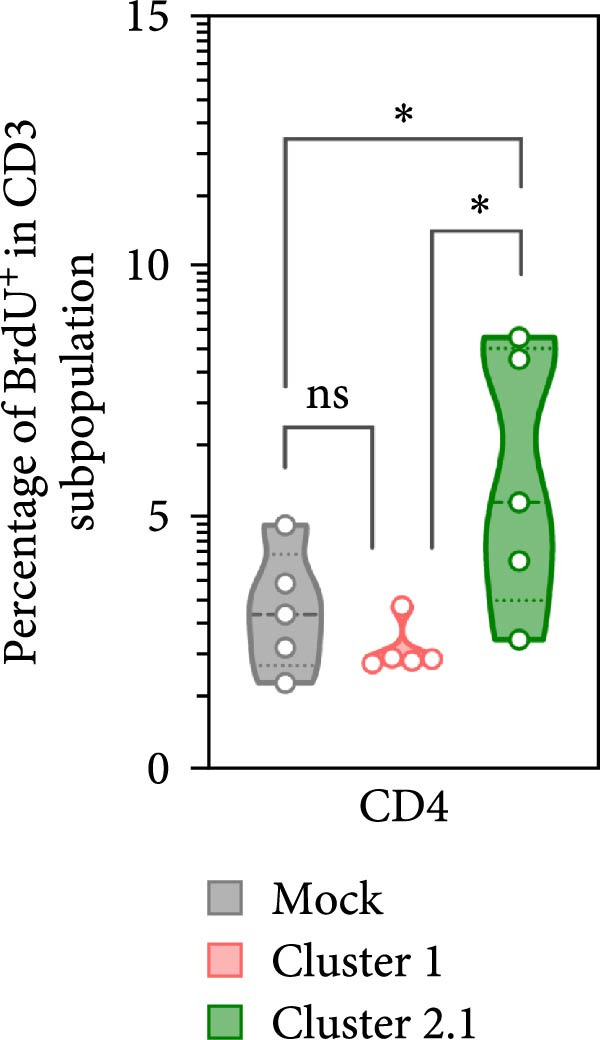
(C)

**Figure:**
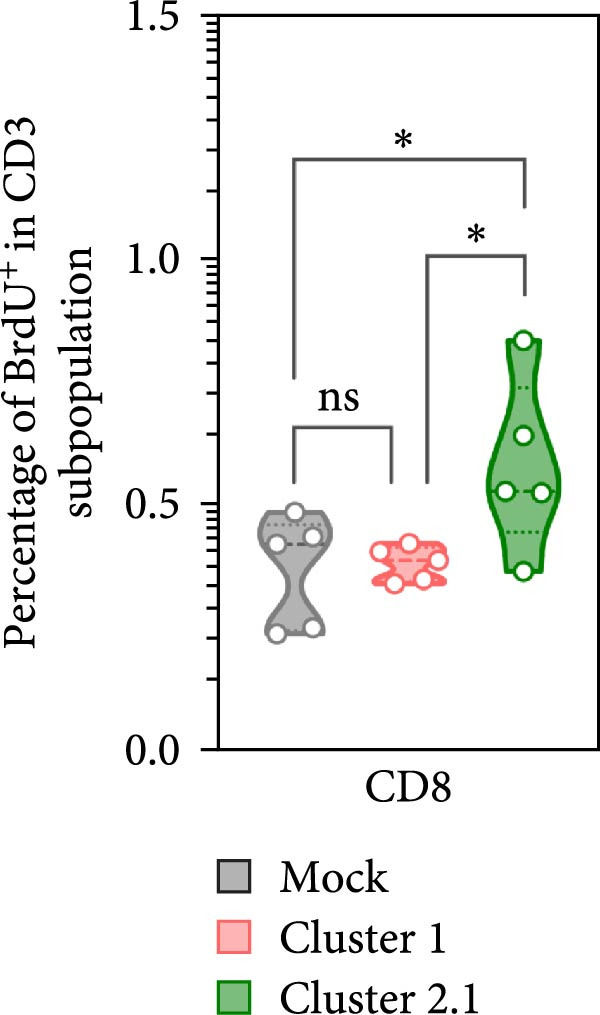
(D)

### 3.4. Increased Proliferation of DTMUV‐Specific T Lymphocytes in Response to Cluster 2.1‐Infected Duck MDM

To further investigate the direct immunomodulatory effects of APCs infected with different DTMUV genotypes on antigen‐specific T lymphocyte activation, duck MDM were initially infected with DTMUV cluster 1 or cluster 2.1 and subsequently co‐cultured with autologous PBL. Proliferating T lymphocyte subsets were then quantified to assess both Th and CTL proliferation (Figure 5A). No significant variation in the frequencies of Th and CTL was observed among the different culture groups (Figure 5B). Consistent with the PBMC findings, co‐culture with cluster 2.1‐infected MDM led to a marked increase in the proliferation of both Th (Figure 5C) and CTL (Figure 5D) subpopulations compared with mock‐infected MDM controls, whereas cluster 1 infection failed to elicit significant responses. These results demonstrate that macrophages infected with different DTMUV genotypes differentially modulate antigen‐specific T lymphocyte activation, with cluster 2.1‐infected macrophages promoting a more robust DTMUV‐specific cellular immune response than cluster 1.

Figure 5DTMUV cluster 2.1‐infected macrophages drive stronger T lymphocyte proliferation in co‐culture. (A) Gating strategy for proliferating T helper (Th; CD3^+^CD4^+^BrdU^+^) and cytotoxic T lymphocytes (CTL; CD3^+^CD8^+^BrdU^+^) in co‐cultures of duck MDM with autologous PBL. (B) No major differences were observed in the overall frequencies of T lymphocyte subpopulations across treatment groups. (C, D) Duck MDM infected with cluster 2.1 significantly enhanced the proliferation of both Th and CTL subpopulations compared with cluster 1 or mock‐infected macrophages. Duck MDM were infected with DTMUV cluster 1, cluster 2.1 (0.1 MOI) or mock and subsequently co‐cultured with autologous PBL (*n* = 5 per treatment) for 72 h. Proliferating T lymphocyte phenotypes were quantified by immunofluorescent staining.  ^∗^ indicates significant differences between treatments.

**Figure:**
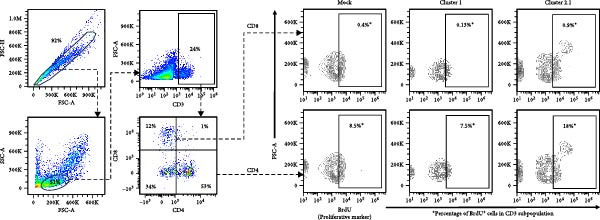
(A)

**Figure:**
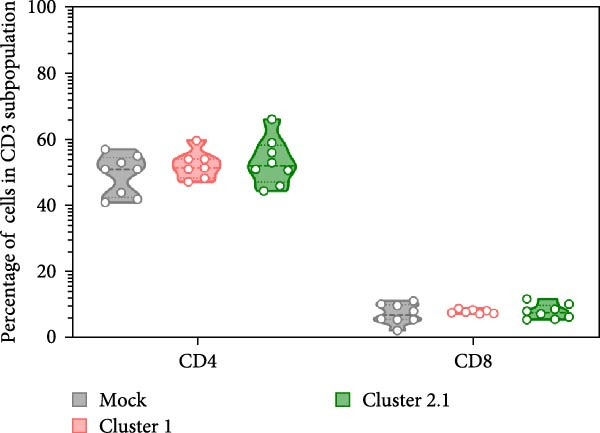
(B)

**Figure:**
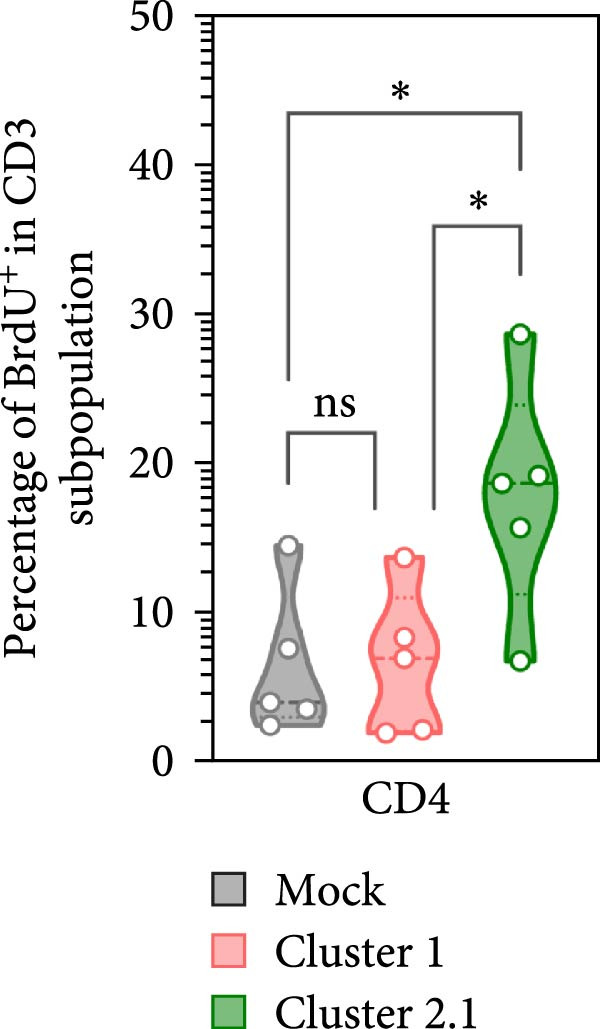
(C)

**Figure:**
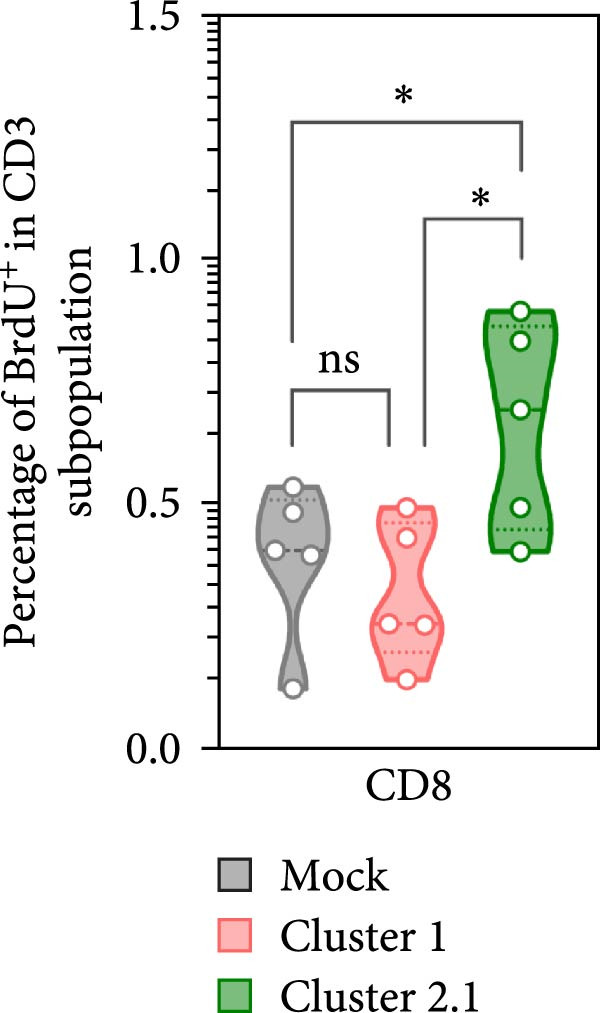
(D)

## 4. Discussion

In the present study, different DTMUV genotypes, including cluster 1 and 2.1, induced distinct levels of apoptosis in duck MDM. Notably, cluster 2.1 inhibited apoptosis and promoted cell survival more effectively than cluster 1. Similar phenomena have been observed in other flaviviruses. For instance, dengue virus has been reported to delay apoptosis by modulating caspase activation and altering Bcl‐2 family protein expression, thereby reducing early apoptotic signaling [24]. Likewise, Japanese encephalitis virus suppresses apoptosis through the PI3K/Akt survival pathway, protecting infected cells from premature cell death [25]. West Nile virus infection can also inhibit apoptosis during the early stages by upregulating host anti‐apoptotic pathways [26]. These previous findings support our observation that infection with DTMUV cluster 2.1 is associated with reduced apoptosis in duck MDM. The reduced apoptosis observed in cluster 2.1‐infected duck MDM may facilitate enhanced viral replication by prolonging host cell survival, thereby allowing extended periods for viral progeny production. This effect could help explain in vivo findings where DTMUV cluster 2.1 infection is associated with earlier viremia, higher viral loads in affected organs, and more severe clinical outcomes in infected ducks compared with cluster 1 [3, 7]. Similar strategies have been reported in other flaviviruses. Dengue virus and Japanese encephalitis virus, for instance, suppress early apoptosis to maintain host cell viability and maximize replication, before inducing apoptosis at later stages to promote viral dissemination [27, 28]. Likewise, West Nile virus has been shown to delay apoptosis in infected cells, resulting in increased viral yield [26]. Collectively, these findings suggest that inhibition of apoptosis represents a conserved mechanism by which flaviviruses enhance replication. The ability of DTMUV cluster 2.1 to suppress apoptosis in duck macrophage may therefore reflect an adaptive trait that confers a replication advantage over DTMUV cluster 1, potentially contributing to its heightened pathogenicity. By inhibiting cell apoptosis, DTMUV cluster 2.1‐infected macrophages may extend their functional lifespan, thereby maintaining continuous production of pro‐inflammatory cytokines and chemokines. Sustained inflammatory signaling is likely to exacerbate immune activation and contribute to tissue pathology in infected ducks. Similar phenomena have been reported in other flaviviruses, including dengue virus infection, which has been shown to delay apoptosis in monocytes, thereby prolonging the production of inflammatory mediators that contribute to vascular leakage and disease severity [29, 30]. Japanese encephalitis virus also modulates macrophage survival, enhancing cytokine secretion and exacerbating neuroinflammation [28, 31]. Taken together, these observations suggest that the inhibition of apoptosis in duck macrophages by DTMUV cluster 2.1 may not only facilitate higher viral replication but also contribute to dysregulated inflammatory responses. This dual effect of enhanced viral replication and heightened inflammation could underlie the more severe clinical signs observed during cluster 2.1 infection, highlighting the role of apoptosis modulation in DTMUV pathogenesis. However, the mechanisms underlying the genotype‐specific differences in apoptosis modulation remain unclear and require further investigation.

In the present study, DTMUV cluster 2.1 induced markedly stronger proliferation of both Th and CTL subsets compared to cluster 1, observed in both the cultured PBMC system and the macrophage–PBL co‐culture system. This observation indicates that different viral genotypes can differentially modulate host cellular immunity. Consistent with previous findings, distinct strains of respiratory syncytial virus (RSV) have been shown to elicit differential immune responses and pulmonary pathophysiology, highlighting the strain‐dependent nature of antiviral immunity [32]. Likewise, the virulence of different vaccinia virus strains is associated with their ability to modulate specific cell‐mediated immune compartments in vivo, demonstrating that viral genetic variations can influence the magnitude and quality of T cell responses [33]. Macrophages are well‐established as efficient APCs that play a central role in priming and amplifying antigen‐specific T lymphocyte responses [34]. One possible explanation for the enhanced duck cellular immunity induced by DTMUV cluster 2.1 is its ability to inhibit apoptosis in infected macrophages. By prolonging the survival of infected APCs, cluster 2.1 may increase the duration of antigen presentation and co‐stimulatory signaling, thereby facilitating more effective induction of antigen‐specific T lymphocyte responses. Supporting this notion, vaccinia virus encodes Bcl‐2‐like anti‐apoptotic proteins that maintain dendritic cell viability, consequently enhancing antigen‐specific T lymphocyte responses [35, 36]. Although DTMUV cluster 2.1 induces a stronger antigen‐specific T lymphocyte response, this cellular immunity does not appear sufficient to confer protection during experimental infection. In infected ducks, viremia and viral loads in the brain are detectable as early as 1 day postinfection (dpi) [3], whereas the peak of virus‐specific T lymphocyte responses is typically observed around 3–5 dpi [37]. Our previous finding suggested that the T lymphocyte response develops too late to effectively control early viral replication and dissemination. These findings highlight a critical limitation in the timing of host cellular immunity relative to the rapid kinetics of DTMUV replication, suggesting that early innate responses, pre‐existing DTMUV‐specific antibodies, or T lymphocytes may play a more decisive role in controlling infection during the initial stages. Therefore, development of DTMUV vaccine that combine early induction of innate immunity with robust antigen‐specific T lymphocyte responses could provide superior protection against natural DTMUV infection.

Furthermore, our findings highlight the critical importance of vaccine strain selection for the development of an effective DTMUV vaccine. Notably, DTMUV cluster 2.1 induced markedly stronger proliferation of both Th and CTL subsets compared to cluster 1, indicating its potential to elicit robust antigen‐specific T lymphocyte responses. Vaccine strains capable of inducing such strong cellular immunity are more likely to confer protective immunity, as observed in other viruses. For example, in influenza A virus, vaccine strains that elicit higher T lymphocyte responses correlate with enhanced viral clearance and reduced clinical disease [38, 39], while in vaccinia virus, strains that stimulate robust CD4^+^ lymphocyte responses provide improved protection upon virulence infection [33]. These data suggest that cluster 2.1 represents a promising candidate for the development of a DTMUV vaccine capable of inducing potent cellular immunity.

In conclusion, our study demonstrates that DTMUV cluster 2.1 markedly inhibits apoptosis in duck macrophages compared to cluster 1, thereby prolonging cell survival. This reduced apoptosis likely facilitates higher viral replication by extending the lifespan of infected cells. Additionally, cluster 2.1‐infected macrophages promote stronger Th and CTL proliferation in both cultured PBMC and macrophage‐PBL co‐culture systems. Together, our findings demonstrate that distinct DTMUV genotypes differentially modulate viral pathogenesis and host immune responses, providing important insights into DTMUV immunopathogenesis. Notably, the ability of cluster 2.1 to elicit robust antigen‐specific T lymphocyte responses highlights its potential as a promising vaccine candidate, underscoring the importance of considering viral genotype in future DTMUV vaccine design.

## Ethics Statement

Animal experiment was approved and conducted in accordance with the ethical guidelines of the Faculty of Veterinary Science, Chulalongkorn University Animal Care and Use Committee (Approval Number 2531061).

## Disclosure

All the authors have read and approved the final manuscript.

## Conflicts of Interest

The authors declare no conflicts of interest.

## Author Contributions

Teerawut Nedumpun designed the study, conducted data collection, statistical analysis, and drafted the manuscript. Kanana Rungprasert and Benchaphorn Limcharoen conducted data collection and statistical analysis. Aunyaratana Thontiravong designed the study and revised the manuscript.

## Funding

This work was supported by the Thailand Science research and Innovation Fund Chulalongkorn University (Grant FOOD_FF_68_246_3100_023).

## Data Availability

The data that support the findings of this study are available from the corresponding author upon reasonable request.
